# Rationale, Design, and Baseline Characteristics of the Prospective Japan Acute Myocardial Infarction Registry (JAMIR)

**DOI:** 10.1007/s10557-018-6839-1

**Published:** 2018-11-23

**Authors:** Satoshi Honda, Kensaku Nishihira, Sunao Kojima, Misa Takegami, Yasuhide Asaumi, Makoto Suzuki, Masami Kosuge, Jun Takahashi, Yasuhiko Sakata, Morimasa Takayama, Tetsuya Sumiyoshi, Hisao Ogawa, Kazuo Kimura, Satoshi Yasuda, on behalf of the JAMIR investigators

**Affiliations:** 1 Department of Cardiovascular Medicine, National Cerebral and Cardiovascular Center, 5-7-1 Fujishiro-dai, Osaka, Suita 565-8565 Japan; 2 Department of General Internal Medicine 3, Kawasaki Medical School, Okayama, Japan; 3 Department of Preventive Medicine and Epidemiologic Informatics, National Cerebral and Cardiovascular Center, Osaka, Japan; 4 Department of Cardiology, Sakakibara Heart Institute, Fuchu, Tokyo Japan; 5 Department of Cardiovascular Medicine, Yokohama City University Medical Center, Yokohama, Japan; 6 Department of Cardiovascular Medicine, Tohoku University, Sendai, Japan

**Keywords:** Acute myocardial infarction, Bleeding event, Antiplatelet therapy

## Abstract

**Background:**

Antiplatelet therapy is a cornerstone of treatment following acute myocardial infarction (AMI). Recently, prasugrel, a new and potent antiplatelet agent, has been introduced in clinical practice. To date, however, real-world in-hospital and follow-up data in Japanese patients with AMI remain limited.

**Objectives:**

To examine ischemic and bleeding events in Japanese patients with AMI and the association between these events and antiplatelet therapy.

**Methods:**

The Japan AMI Registry (JAMIR) is a multicenter, nationwide, prospective registry enrolling patients with AMI from 50 institutions. The inclusion criterion is spontaneous onset of AMI diagnosed based on either the universal definition or Monitoring Trends and Determinants in Cardiovascular disease (MONICA) criteria. The major exclusion criteria are hospital admission ≥ 24 h after onset, no return of spontaneous circulation on admission following out-of-hospital cardiopulmonary arrest, and AMI as a complication of percutaneous coronary intervention or coronary artery bypass grafting. The primary end point of the study is the composite of cardiovascular death, non-fatal myocardial infarction, and non-fatal stroke. Major safety end points include major bleeding based on Thrombolysis in Myocardial Infarction (TIMI) criteria and type 3 or type 5 bleeding based on Bleeding Academic Research Consortium (BARC) criteria. Between December 2015 and May 2017, a total of 3411 patients (mean age 68.1 ± 13.2 years, 23.4% female) were enrolled in the study. Patients will be followed for 1 year.

**Conclusions:**

JAMIR will provide important information regarding contemporary practice patterns in the management of Japanese patients with AMI, their demographic and clinical characteristics, in-hospital and post-discharge outcomes, and how they are related to antiplatelet therapy.

**Electronic supplementary material:**

The online version of this article (10.1007/s10557-018-6839-1) contains supplementary material, which is available to authorized users.

Despite major advances in prevention and treatment, cardiovascular disease is the second leading cause of mortality in Japan. It continues to be a major public health burden, and its prevalence is expected to increase with an increasing aging society and lifestyle changes [1, 2]. Among cardiovascular diseases, acute myocardial infarction (AMI) is a major cause of mortality and disability.

In the past few decades, various types of studies on AMI have been carried out to improve treatment [3, 4]. Most of the evidence has been provided by studies carried out in Western countries [4]. However, Japanese individuals have different physiques, genetic backgrounds, and medical environments [5]. It is unknown whether evidence from Western countries could be generalized to clinical practice in Japan. Consequently, the Japan Acute Myocardial Infarction Registry (JAMIR) was initiated to generate original data about Japanese subjects. Recently, a retrospective analysis was performed [6].

Antiplatelet therapy is a cornerstone of treatment to prevent thrombotic complications after AMI [7, 8]. Dual antiplatelet therapy (DAPT) with aspirin and clopidogrel is the conventional gold standard for antiplatelet therapy after AMI and coronary stenting. However, clopidogrel exhibits significant interpatient variability in antiplatelet response, mainly because of genetic variations in cytochrome P450 (CYP) 2C19 [9]. Of note, decreased response to clopidogrel is common among Asians due to a high prevalence of genetic polymorphisms [10–12]. Indeed, the prevalence of CYP2 C19*3 allelic variants, a major loss-of-function polymorphism in CYP2C19, is 24.2% among Japanese, 14.8% among Koreans, but only 0.4% among Africans and 0.2% among Caucasians [11].

Recently, prasugrel has been introduced as a new potent antiplatelet agent. Prasugrel is expected to be a better antiplatelet agent than clopidogrel because it exerts its antiplatelet effect more rapidly, and there are fewer individual differences in metabolism [13, 14]. In the TRITON-TIMI 38 trial, the safety and efficacy of prasugrel and clopidogrel were compared in patients with acute coronary syndrome (ACS) [15]. At a median follow-up of 14.5 months, the incidence of ischemic events with a 60-mg loading dose and a 10-mg maintenance dose of prasugrel was 19% lower than with clopidogrel (prasugrel arm, 9.9% vs. clopidogrel arm, 12.1%). Importantly, subanalyses of TRITON-TIMI 38 trial demonstrated that common functional CYP genetic variants were associated with diminished platelet inhibition and higher rates of cardiovascular events in patients treated with clopidogrel but not in those treated with prasugrel [16, 17]. However, the incidence of bleeding events was 32% higher in the prasugrel group (prasugrel arm, 2.4% vs. clopidogrel arm, 1.8%). Since the TRITON-TIMI 38 trial was conducted in 30 Western and South American countries, but no Asian countries, it is unclear whether these results can be simply extrapolated to Asian populations. Asian populations have a lower risk of ischemic events and a higher risk of bleeding events than Western populations [18–20]. Therefore, it is possible that a lower level of platelet inhibition would be sufficient in Asian populations than in Western populations. In this context, the PRASFIT-ACS study was carried out to establish the appropriate dosage of prasugrel for Japanese patients with ACS. In the trial, a 20-mg loading dose and a 3.75-mg maintenance dose of prasugrel were compared with conventional doses of clopidogrel. At 24 weeks of follow-up, the incidence of major adverse cardiovascular events was numerically lower in the prasugrel group than in the clopidogrel group (9.4% vs. 11.8%). There was no apparent increase in the incidence of bleeding events (1.9% vs. 2.2%). Based on these results, a loading dose of 20 mg and a maintenance dose of 3.75 mg were established for prasugrel in Japan. Prasugrel was approved in 2014 in Japan. It has been used in the treatment of patients with ACS [21]. However, real-world clinical data on Japanese patients with AMI remain limited. Therefore, the present JAMIR study was designed to prospectively assess ischemic and bleeding events in Japanese patients with AMI and their association with antiplatelet therapy.

## Methods

### Study Population

**Fig. 1:**
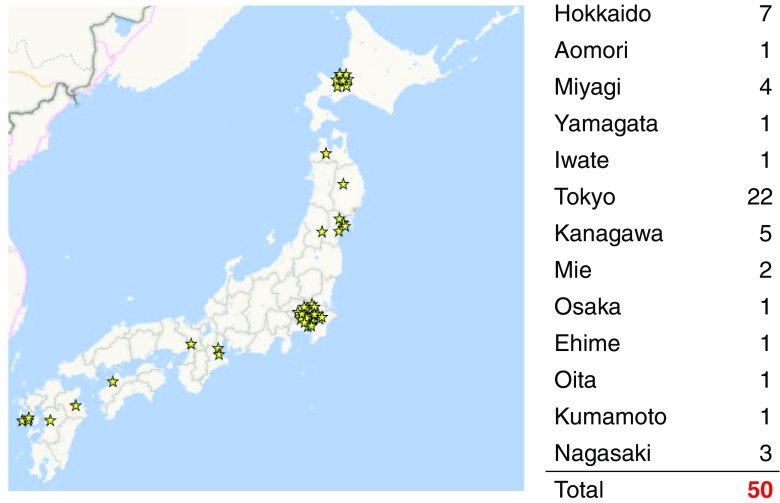
Number of participating institutions by location (prefecture)

We registered all consecutive patients presenting with spontaneous onset of AMI between December 2015 and May 2017 at 50 institutions (Fig. 1). Table 1 summarizes the registry’s inclusion and exclusion criteria. AMI was diagnosed based on either the MONICA criteria or the universal definition [22, 23]. We excluded patients who were admitted to the hospital ≥ 24 h after onset, with no return of spontaneous circulation on admission after out-of-hospital cardiopulmonary arrest, or had AMI as a complication of percutaneous coronary intervention (PCI) or coronary artery bypass grafting (CABG). Management of patient was dependent on the discretion of treating physician, and antiplatelet regimen and dosage were determined according to the guidelines of the Japanese Circulation Society [24, 25].

**Table 1:** Inclusion and exclusion criteria

Inclusion criterion	
AMI diagnosed based on either MONICA criteria or the universal definition	
Exclusion criteria	
• Admission ≥ 24 h after onset	
• Out-of-hospital cardiopulmonary arrest with no return of spontaneous circulation on admission	
• AMI as a complication of PCI or CABG	

*AMI* acute myocardial infarction, *PCI* percutaneous coronary intervention, *CABG* coronary artery bypass grafting

### Study End Points

The primary end point of the study is the composite of cardiovascular death, non-fatal myocardial infarction, and non-fatal stroke. Cardiovascular death is defined as any death with a demonstrable cardiovascular cause or any death that is not clearly attributable to a non-cardiovascular cause. Myocardial infarction must be distinct from the index event and is defined based on symptoms suggestive of ischemia or infarction, electrocardiographic data, cardiac biomarker data, or pathological evidence of infarction depending on the clinical situation based on criteria adapted from the American College of Cardiology definitions (Bethesda, MD) [26, 27]. Major safety end points include major bleeding based on the Thrombolysis in Myocardial Infarction (TIMI) criteria and type 3 or 5 bleeding based on the Bleeding Academic Research Consortium (BARC) criteria [28, 29].

Secondary end points include a composite of ischemic events (cardiovascular death, non-fatal myocardial infarction, and non-fatal cerebral infarction) and bleeding events (major bleeding based on TIMI criteria); individual components of ischemic events; all-cause death; stent thrombosis; major and minor bleeding based on TIMI criteria; type 2, 3, or type 5 bleeding based on the BARC criteria; and hospital readmission due to heart failure. Stent thrombosis was defined as definite or probable stent thrombosis according to the Academic Research Consortium definition [30].

### Data Collection

All patients who met the eligibility criteria were registered at each study site. Primary data collection is derived from the patient’s medical record. Information was collected on patient demographics, medical history, ambulance use, details about coronary angiography and invasive therapy, cardiac medications, and outcomes (Table 2). Investigators, clinical research coordinators, or local data managers at each study site registered the data using the JAMIR registration system. The data were anonymized in a linkable manner at each study site before they were sent. A follow-up study of patients was conducted 1 year after the onset of AMI based on the medical information available at each study site. A letter requesting for follow-up was sent to patients whose medical information was not available at the study sites after 1 year due to hospital transfer or other reasons. When the letter was sent, appropriate informed consent was obtained.

**Table 2:** Variables in JAMIR

Clinical demographics	Age, sex, height, weight, ambulance use, presence of out-of-hospital cardiac arrest, date of admission, STEMI or NSTEMI, Killip class, systolic and diastolic blood pressure on admission, heart rate on admission, presence of hypertension, presence of diabetes, presence of dyslipidemia, smoking, previous myocardial infarction, history of CABG, history of PCI, history of atrial fibrillation, history of cerebrovascular disease, history of malignancy, history of peripheral artery disease
Invasive procedures	Use of emergency CAG, approach site, culprit lesion site, number of diseased vessels, use of reperfusion therapy (PCI or thrombolytic therapy), use of stenting, stent type (drug-eluting stent or bare metal stent), date of reperfusion, door-to-device time, TIMI classification on final angiography, concurrent PCI in non-culprit lesion, use of IABP, use of V-A ECMO, use of CABG
Test results during hospitalization	Peak serum CK, peak serum CK-MB, peak troponin T, serum creatinine, hemoglobin on admission, blood glucose on admission, total cholesterol, LDL cholesterol, HDL cholesterol, triglycerides, LVEF on echocardiography
Medications during hospitalization	Antiplatelet agents (loading and maintenance doses), ACE inhibitors, ARBs, β-blockers, statins, anticoagulants (warfarin or DOAC), proton pump inhibitor
Complications during hospitalization	Cardiogenic shock, cardiac rupture, ventricular septal perforation, acute mitral regurgitation (papillary muscle rupture), right ventricular involvement
Medications after discharge	Antiplatelet agents, statins, anticoagulants
Test results during follow-up	Total cholesterol, LDL cholesterol, HDL cholesterol, triglycerides
Outcomes	Cardiovascular death, non-cardiovascular death, non-fatal myocardial infarction, non-fatal cerebral infarction, major and minor bleeding based on TIMI criteria, type 2, 3, or type 5 bleeding based on BARC criteria, stent thrombosis, readmission due to heart failure

*JAMIR* Japan Acute Myocardial Infarction Registry, *STEMI* ST elevation myocardial infarction, *NSTEMI* non–ST elevation myocardial infarction, *CABG* coronary artery bypass grafting, *PCI* percutaneous coronary intervention, *CAG* coronary angiography, *IABP* intra-aortic balloon pumping, *V-A ECMO* venoarterial extracorporeal membrane oxygenation, *CK* creatine kinase, *LDL* cholesterol low-density lipoprotein cholesterol, *HDL* cholesterol high-density lipoprotein cholesterol, *LVEF* left ventricular ejection fraction, *ACE* angiotensin-converting enzyme, *ARB* angiotensin receptor blocker, *DOAC* direct oral anticoagulant, *TIMI* Thrombolysis in Myocardial Infarction, *BARC* Bleeding Academic Research Consortium

### Data Analysis

Univariate and multivariate analyses will be performed to identify determinants of ischemic and bleeding events after AMI. All pre-specified and exploratory analyses will be performed by the JAMIR group using SAS (SAS Institute, Inc., Cary, NC, USA).

Baseline continuous variables will be presented as means ± SD or medians and interquartile range, depending on the distribution of the data. Categorical variables will be presented as counts and percentages.

### Study Organization

This prospective study is planned by the Japan Cardiovascular Research Foundation and is coordinated by the JAMIR group. IQVIA Services Japan serves as the contract research organization and provides data and site management services. The steering committee is responsible for the scientific content of the protocol, protocol implementation, results presentation, and manuscript preparation. Trial operations are monitored and coordinated by the operation committee.

### Baseline Patient Characteristics

**Fig. 2:**
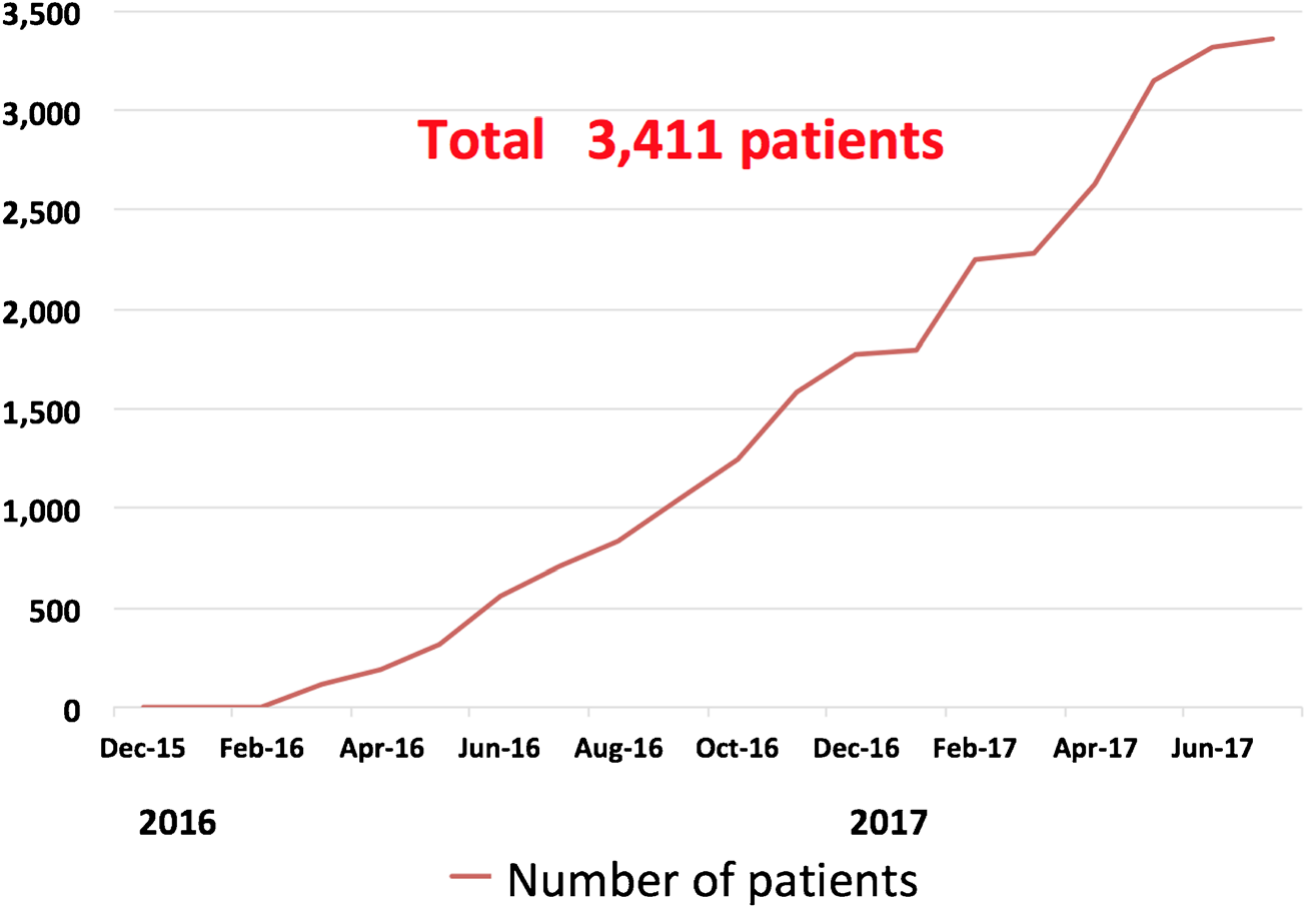
Number of patients enrolled in Japan Acute Myocardial Infarction Registry

JAMIR started enrollment in December 2015. By the end of July 2017, a total of 3411 patients were registered from 50 sites across Japan (Fig. 2). All institutes except one used the universal definition for the diagnosis of AMI, and 99% of study patients (3371 of 3411) were diagnosed by the universal definition. Patient characteristics are presented in Table 3. The mean age was 68.1 ± 13.2 years, and 23.4% was female. ST elevation myocardial infarction (STEMI) accounted for 77% of patients with AMI. With respect to infarct location, anterior was the most frequent (51%), followed by inferior (41%), and lateral or posterior (10%). The prevalence of coronary risk factors was 75.1% for hypertension, 38.7% for diabetes, and 71.7% for dyslipidemia. Ten percent of patients had a prior myocardial infarction, 8.8% of patients had a history of atrial fibrillation, and 9.9% of patients had malignancy. Of note, 97% of patients underwent emergent coronary angiography and primary PCI defined as emergent or urgent PCI was performed in 93% of patients overall. During hospitalization, almost all patients were treated with aspirin (97.2%). The most frequent P2Y12 inhibitor used was prasugrel (80.6%), followed by clopidogrel **(**17.2%). Warfarin and direct oral anticoagulants (DOACs) were administered to 6.2% and 7.4% of patients, respectively. With respect to the dose of prasugrel, 96.2% of patients received 3.75 mg (Table 4).

**Table 3:** Baseline characteristics (*n* = 3411)

Age (years)	68.1 ± 13.2
Gender, female (%)	23.4
STEMI (%)	77.0
Killip class ≥ II (%)	23.3
Hypertension (%)	74.4
Diabetes (%)	36.2
Dyslipidemia (%)	70.7
Prior stroke (%)	9.9
Prior myocardial infarction (%)	9.7
PAD (%)	4.3
Prior CABG (%)	2.5
Prior PCI (%)	11.6
Atrial fibrillation (%)	6.9
Malignancy (%)	8.7
Current smoking (%)	41.9
Medication during hospitalization (%)	
Antiplatelet therapy	
Aspirin	97.2
Prasugrel	80.6
Clopidogrel	17.2
Ticlopidine	0.3
Warfarin	6.2
DOAC	7.4
Emergent coronary angiography (%)	97.0
Primary PCI (%)	93.2
Thrombolysis (%)	0.2

Primary PCI is defined as the emergent or urgent use (within 24 h after admission) of PCI

*STEMI* ST elevation myocardial infarction, *PAD* peripheral artery disease, *CABG* coronary artery bypass grafting, *PCI* percutaneous coronary intervention, *DOAC* direct oral anticoagulant

**Table 4:** Maintenance doses of antiplatelet drugs

	Maintenance dose	Percent
Aspirin	100 mg	95.5
	81 mg	3.2
	Other	1.3
Prasugrel	3.75 mg	96.2
	Other	3.8
Clopidogrel	75 mg	95.1
	Other	4.9

## Discussion

JAMIR is a registry of Japanese patients with AMI designed to be a multicenter, nationwide, prospective observational study with the primary objective of examining ischemic and bleeding events and their relationship with antiplatelet therapy. A total of 3411 patients were enrolled between December 2015 and May 2017; soon after prasugrel, a potent antiplatelet agent, was introduced in Japan.

The Prevention of Atherothrombotic Incidents Following Ischemic Coronary Attack (PACIFIC) registry is a multicenter, prospective, observational study of Japanese patients with ACS [31]. Between May 2008 and May 2009, 3597 consecutive patients aged ≥ 20 years hospitalized for ACS were enrolled at 96 hospitals and followed for 2 years. The majority (93.5%) of patients underwent PCI, with a success rate of 93.9%. Antiplatelet agents were prescribed to 99.3% of patients, and 92.6% of the entire group received DAPT (e.g., aspirin plus clopidogrel). The cumulative incidence of major adverse cardiac and cerebrovascular events (MACCEs) was 6.4%, and all-cause mortality was 6.3%. However, due to pharmacological limitations of clopidogrel (interindividual variability in biological efficacy, slow onset of action, mild inhibition of platelet reactivity), ischemic recurrences remained common following stent implantation, especially in patients with ACS. Thus, more potent P2Y12 inhibitors, such as prasugrel and ticagrelor, were developed to overcome these pitfalls. New P2Y12 inhibitors have a considerable safety and tolerability profile and are associated with lower mortality in patients undergoing PCI compared with clopidogrel; however, the risk/benefit ratio of ischemic and bleeding events should be further investigated in real-world clinical practice.

Indeed, the recent Korean AMI Registry (KAMIR) study demonstrated that prasugrel and ticagrelor had similar rates of MACCEs at 1 year, but a higher rate of bleeding events compared with clopidogrel (MACCEs, clopidogrel 2.1% vs. prasugrel 2.6% vs. ticagrelor 2.1%, *p* = 0.790; bleeding events, clopidogrel 3.1% vs. prasugrel 8.0% vs. ticagrelor 8.0%, *p* < 0.001) [32]. However, it should be noted that Korean patients who underwent PCI received 300 mg aspirin and 60 mg prasugrel or 180 mg ticagrelor as a loading dose prior to PCI. The maintenance dose after PCI was 100–300 mg aspirin and/or 5 or 10 mg prasugrel once daily or 90 mg ticagrelor twice daily [33]. Despite having an East Asian study population, this study included doses of prasugrel that are different from that of Japan; thus, further studies like the present JAMIR study are warranted to adapt Western guidelines on new potent P2Y12 inhibitors for East Asians.

With regard to the definition of AMI, MONICA criteria had been widely used in Japan [23]. The first universal definition was developed in 2000 [34], and the third universal definition was published in 2012 [22]. Although the universal definition based on high-sensitive troponin measurement was becoming common in Japan, we did not know how much participating hospital applied the universal definition when we planned the JAMIR project. Accordingly, we allowed both definitions for the JAMIR; however, as a result, all institutes except for one used the third universal definitions. This result suggests that diagnosis of AMI based on high-sensitive troponin measurement has become common in Japan.

## Conclusion

This study will provide important information regarding contemporary practice patterns in the management of Japanese patients with AMI, their demographic and clinical characteristics, and in-hospital and post-discharge outcomes.

## Electronic Supplementary Material

ESM 1 (DOCX 209 kb)
