# Supplementary material for: Rationale, Design, and Baseline Characteristics of the Prospective Japan Acute Myocardial Infarction Registry (JAMIR)
Source: Cardiovasc Drugs Ther. 2018 Nov 23;33(1):97–103. doi: 10.1007/s10557-018-6839-1 (PMC6433805; doi:10.1007/s10557-018-6839-1)
Supplement: Supplementary file 1 — (DOCX 209 kb) [file 10557_2018_6839_MOESM1_ESM.docx]

**Definitions used in the JAMIR**

**Past history**

Diabetes^1^:

At least one of following:

- Fasting blood glucose ≥ 126 mg/dL
- Casual blood glucose ≥ 200 mg/dL
- HbA1c ≥ 6.5%
- 2 hour OGTT (75 g oral glucose tolerance test) glucose value ≥200 mg/dL
- Current use of antidiabetic medication

# 1) Diagnosis and classification of diabetes mellitus. Diabetes Care 2009; 32: 1327–1334.

Hypertension^2:^

At least one of following:

- Systolic BP ≥ 140 mmHg
- Diastolic BP ≥ 90 mmHg
- Current use of antihypertensive medication

2) The Japanese Society of Hypertension Guidelines for the Management of Hypertension (JSH 2014). Hypertens Res 2014; 37: 253–392.

Dyslipidemia^3^:

At least one of following:

- Total cholesterol ≥ 220 mg/dL
- LDL-cholesterol ≥ 140 mg/dL
- HDL-cholesterol ≥ 40 mg/dL
- Fasting triglycerides ≥ 150 mg/dL
- Current use of a lipid-lowering agent

# 3) Executive summary of the Japan Atherosclerosis Society (JAS) guidelines for the diagnosis and prevention of atherosclerotic cardiovascular diseases in Japan, 2012 version. J Atheroscler Thromb 2013; 20: 517–523.

Atrial fibrillation (AF):

History of AF, including both paroxysmal and persistent AF.

Stroke:

History of stroke includes TIA, ischemic stroke, cerebral hemorrhage, and subarachnoid hemorrhage

Smoking:

Current smoking or history of smoking within the past year

Peripheral artery disease:

Significant stenosis (≥50%) in a peripheral artery (renal, common iliac, femoral, etc.) or at least one of following:

・Intermittent claudication

・ABI ≤ 0.9

・History of amputation, bypass surgery, or percutaneous intervention due to stenosis or occlusion of a peripheral artery

**Clinical outcomes**

Myocardial infarction (MI):

Myocardial infarction must be distinct from the index event. It is defined based on symptoms suggestive of ischemia or infarction, electrocardiographic data, cardiac biomarker data, or pathologic evidence of infarction depending on the clinical situation, using the following criteria:


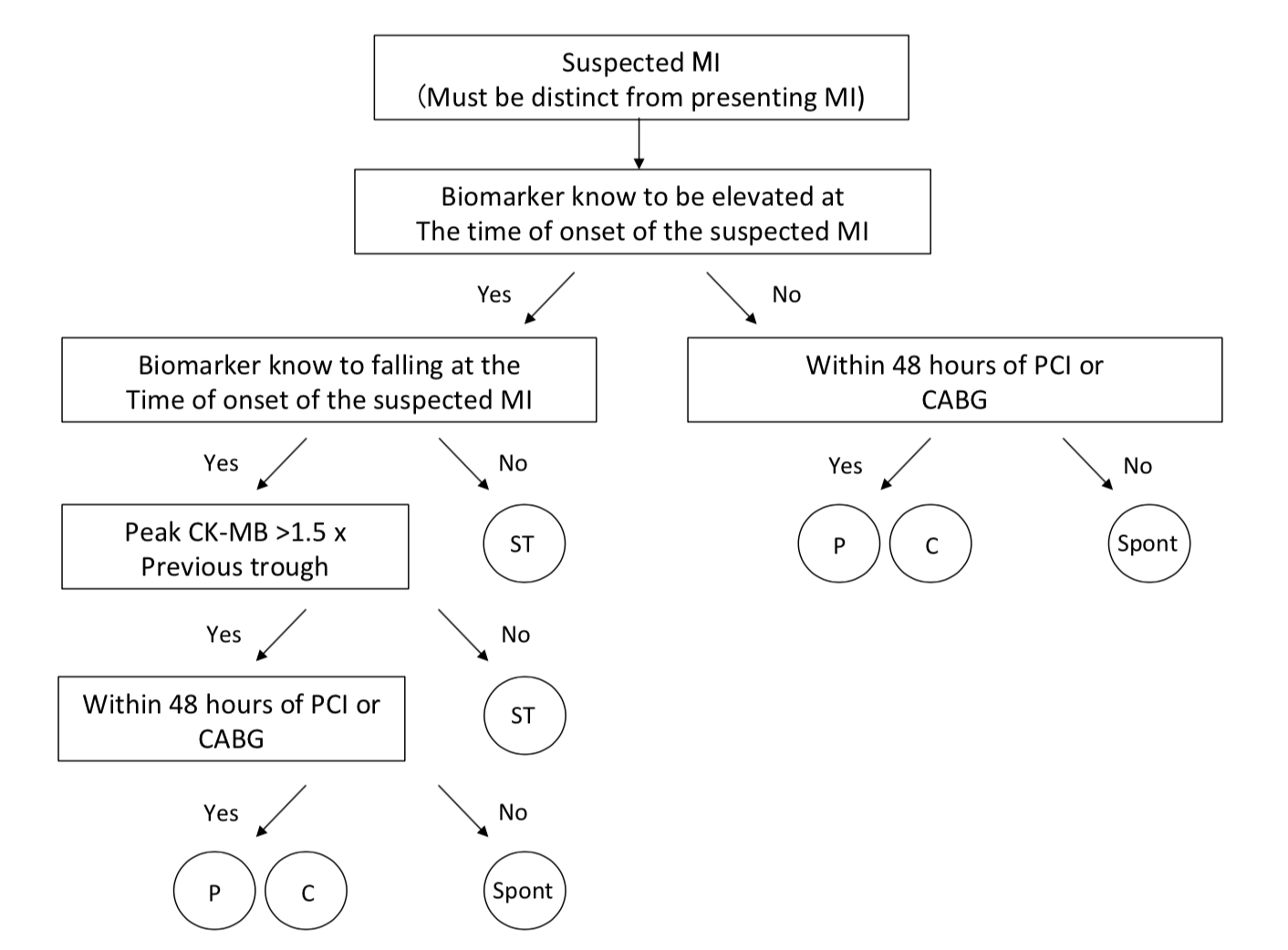


**Five major sets of criteria will be used for the diagnosis of nonfatal MI.**

- ST= elevation or reelevation of ST segment and one of the following:
- Ischemic chest pain or equivalent longer than 20 minutes.
- Hemodynamic decompensation.
- Spont = spontaneous: CK-MB or troponin greater than the ULN and one of the following:
- Ischemic chest pain (or angina equivalent) greater than 20 minutes
- ST-segment deviation 1mm or more in one or more leads
- P = PCI: CK-MB greater than 3 times ULN on 2 samples post-PCI, or greater than 5 times ULN on 1 sample, provided it is the final sample and is greater than 12 hours after PCI.
- C = CABG: CK-MB greater than 10 times ULN on 1 sample after CABG.
- New Q waves 0.04 seconds or longer, or pathology distinct from prior MI.

Adapted from Am Heart J 2006;152: 627–35. Evaluation of prasugrel compared with clopidogrel in patients with acute coronary syndromes: design and rationale for the TRial to assess improvement in Therapeutic Outcomes by Optimizing Platelet Inhibition with Prasugrel Thrombolysis on Myocardial Infarction 38 (TRITON-TIMI 38)

Cerebral infarction:

Acute neurological finding or symptom along with CT or MRI documentation

Stent thrombosis:

Definite

Confirmation of stent thrombosis by angiography

A thrombus derived from a region inside the stent or within 5 mm proximal or distal to the stent and presence of at least one of the following within a 48-hour time window :

- Acute onset of ischemic symptoms at rest
- New development of ischemic electrocardiographic changes suggestive of acute ischemia
- A typical increase and decrease in cardiac biomarkers
- Nonocclusive thrombus
- Occlusive thrombus

Confirmation of stent thrombosis by pathology

Histological findings of subacute in-stent thrombus after autopsy or thrombectomy

Probable

Probable stent thrombosis is considered to have occurred after intracoronary stenting

in the following cases:

- - - Unexplained death within 30 days
    - Myocardial infarction related to acute ischemia after stent implantation in which angiography revealed no stent thrombosis and no other cause of death can be identified any time after stent implantation

TIMI bleeding:

Minor bleeding (present/absent):

Presence of clinically obvious signs of bleeding with a fall in hemoglobin concentration of 3–5 g/dL.*

Major bleeding (present/absent)

- Intracranial hemorrhage
- Presence of obvious clinically serious signs of bleeding with a fall in hemoglobin concentration of >5 g/dL.*

Note: Bleeding related to CABG is not included.

*If a blood transfusion is given, hemoglobin concentration should be adjusted by the number of packed red blood cells given the baseline and post-transfusion measurements. For this calculation, it is assumed that a transfusion of 1 unit of hemoglobin increases hemoglobin concentration by 1 g/dL. Therefore, the change in hemoglobin is calculated using the following formula:

∆ hemoglobin = [baseline hemoglobin level − post-transfusion hemoglobin level] + [number of units transfused]

BARC bleeding criteria:

Type 2

Presence of obvious signs of bleeding that should be medically treated. While the criteria for type 3, type 4, and type 5 criteria are not met, at least one of the following criteria: (1) non-surgical intervention by a medical professional is required; (2) hospitalization or an increase in the level of care is required; (3) assessment is required.

Type 3

Type 3a

Obvious bleeding and a fall in hemoglobin concentration of 3–5 g/dL

Blood transfusion for obvious bleeding

Type 3b

Obvious bleeding and a fall in hemoglobin concentration ≥5 g/dL

Cardiac tamponade

Bleeding requiring surgical intervention (excluding dental, nasal, skin, and hemorrhoidal bleeding)

Bleeding requiring intravenous injection of vasoactive drugs

Type 3c

Intracranial hemorrhage (not including microhemorrhage or hemorrhagic changes, but

including intraspinal hemorrhage)

Subcategory confirmed by autopsy, diagnostic imaging, or lumbar puncture

Intraocular hemorrhage with impaired visual acuity

Type 4

CABG-related bleeding: not included as an event in this study

Type 5 (Fatal bleeding)

Type 5a

Bleeding highly likely to be fatal: not confirmed by autopsy or imaging, but clinically suspected

Type 5b

Obviously fatal bleeding

In-hospital complications

Cardiogenic shock:

Systolic blood pressure of <90 mmHg for more than 30 min or requiring infusion of catecholamines to maintain a systolic pressure >90 mmHg, and impaired end-organ perfusion. The diagnosis of impaired end-organ perfusion required at least one of the following: altered mental status; cold, clammy skin and extremities; oliguria with urine output of <30 mL/h; or serum lactate level > 2.0 mmol/L.

Acute free wall rupture:

Abrupt transmural rupture of the infarct area, causing hemopericardium and death in <30 min

Subacute free wall rupture:

Gradual or incomplete rupture of the infarct area with slow or recurrent bleeding into the pericardial sac, causing progressive or recurrent cardiac tamponade

Right ventricular involvement:

AMI with at least one of following:

- ST elevation in V4R ≥ 0.1 mV
- Right ventricular enlargement or akinesis demonstrated by echocardiography
- At least one of the following hemodynamic criteria for right ventricular involvement:
- Mean right arterial pressure of ≥10 mmHg and <5 mmHg difference from mean pulmonary capillary wedge pressure
- Noncompliant right atrial pressure waveform pattern
- Pulmonary pulsus alternans or early rising pulmonary artery pressure
